# Impact of ambient air pollution and socio-environmental factors on the health of children younger than 5 years in India: a population-based analysis

**DOI:** 10.1016/j.lansea.2023.100328

**Published:** 2023-12-02

**Authors:** Paul E. George, Nandan Thakkar, Sandul Yasobant, Deepak Saxena, Jay Shah

**Affiliations:** a Emory University, School of Medicine, Atlanta, GA, USA; b Emory University, Rollins School of Public Health, Atlanta, GA, USA; c University of North Carolina, School of Medicine, Chapel Hill, NC, USA; d Indian Institute of Public Health Gandhinagar, Gujarat, India; e School of Epidemiology & Public Health, Datta Meghe Institute of Medical Sciences, Wardha, India; f Children's Healthcare of Atlanta, Atlanta, GA, USA; g Aflac Cancer and Blood Disorders Center, Children's Healthcare of Atlanta, Atlanta, GA, USA

**Keywords:** India, Air pollution, Child, Under-5, Social determinants of health, Environmental determinants of health

## Abstract

**Background:**

Ambient air pollution and household environmental factors affect child health, particularly in low-income and middle-income countries. This study aimed to investigate the association between ambient air pollution (PM2·5) levels, socio-environmental factors (including household wealth, housing quality measures, smoking status), and the occurrence of respiratory illness in Indian children.

**Methods:**

In this retrospective and observational study, we analysed data from India's National Family Health Survey (NFHS-5, 2019–2021) combined with NASA's Global Annual PM2·5 Grids database. Bivariate and multivariable generalized additive models were employed to examine associations between key social-environmental factors and respiratory illness in children younger than 5 years.

**Findings:**

We analysed data from 224,214 children younger than 5 years, representing 165,561 families from 29,757 geographic clusters. Our results showed extremely high annual PM2·5 levels throughout India (median 63·4·g/m^3^, IQR 41·9–81·6), with higher exposure for rural and impoverished families. In bivariate analyses, PM2·5 was significantly associated with reported respiratory illness (p < 0·001). Using generalized additive models and after accounting for key social and environmental factors, a monotonic increasing and non-linear relationship was observed between PM2·5 and respiratory illness (p < 0·001), with increased likelihood of illness observed even at values near and below India's National Ambient Air Quality Standards of 40 μg/m^3^.

**Interpretation:**

The study highlights the significant association of social-environmental conditions with health outcomes among young children in India. Efforts specifically targeting ambient air pollution and child health during monsoon season could have significant health benefits among this population and help achieve the goal of ending preventable deaths of children younger than 5 years.

**Funding:**

10.13039/100000002National Institutes of Health (NIH T-32-HL139443-3).

**Research in context**

**Evidence before this study**

India experiences high comparative rates of ambient (outdoor) air pollution, childhood poverty, in addition to morbidity and mortality in children younger than 5 years (under-5). We searched PubMed and Google Scholar for articles published up to Dec 31, 2022, using the search terms “ambient air pollution” AND “India” AND (“child” OR “pediatric” OR “under-5”). No language or time restrictions were applied to this search. Although studies have documented the detrimental health effects of ambient air pollution in India, they have primarily focused on urban settings and relied on local pollution monitors. Nationwide studies using remote-sensing data have found associations between child health and air pollution, though the spatial resolution of these studies was >10 square km. Consequently, these studies have not fully captured the considerable geographic variability of ambient air pollution or addressed the rural population, where a significant portion of under-5 morbidity and mortality occurs in India. Additionally, these studies have often neglected other crucial social and environmental determinants of health, such as household sources of pollution, household sanitation, and family wealth.

**Added value of this study**

Our nationwide analysis of 224,214 children under-5 in India offers a comprehensive assessment of the impact of ambient air pollution and socio-environmental factors on recent respiratory illness in this vulnerable population. By combining nationwide data from India's National Family Health Survey (NFHS-5) with novel remote-sensing air pollution data across India, we uncovered the significant association of ambient air pollution with severe respiratory disease in Indian children under-5. Our study demonstrates that ambient air pollution is a widespread issue, not confined to urban areas, and that pollution mitigation efforts might have significant benefits for respiratory illness, the major driver of morbidity and mortality in this age group. Importantly, our investigation considered multiple social and environmental risk factors, offering a comprehensive understanding of the complex associations between social, economic, and environmental factors affecting under-5 child health outcomes.

**Implications of all the available evidence**

Our study finds that high levels of air pollution in India, including in rural regions, are significantly associated with respiratory illness in young children, a significant source of childhood morbidity and mortality. Although reducing pollution values to the Indian standard of PM2·5 40 μg/m³ would be an improvement, our results indicate that even at this level, significant early child morbidity may persist. These findings emphasise the need for targeted interventions aimed at reducing ambient air pollution and ensuring special attention is given to under-5 health during the monsoon season. Such interventions could yield important benefits for children under-5 and help achieve the Sustainable Development Goal (SDG) Target 3·2: End preventable deaths of newborns and children under 5 years of age.

## Introduction

The environment in which one is born and lives contributes significantly towards health and well-being and is a well-established social determinant of health (SDoH).^1^ A World Health Organization (WHO) study estimated that 23% of all deaths globally are attributable to the environment and that up to 26% of all deaths of children younger than 5 years (under-5) could be avoided if environmental risks were eliminated.^2^ Air pollution has garnered the specific attention of health researchers over the past decades and is now estimated to be responsible for greater than 6 million deaths per year worldwide, with two-thirds attributed to ambient (outdoor) air pollution and one-third to household air pollution in 2015.^3^ Furthermore, rapidly industrialising low-income and middle-income countries are disproportionately impacted by air pollution and the majority of life-years lost due to air pollution occur in these countries.^4^

India has disproportionately high morbidity and mortality due to air pollution. A 2019 study estimated the annual population-weighted mean exposure to PM2·5—fine particulate matter 2·5 microns or less in diameter, the most well-characterised pollutant with regards to human health—in India at 90 μg/m^3^, which far exceeds both the WHO Annual Air Quality Guideline level of 5 μg/m^3^ and India's National Ambient Air Quality Standards of 40 μg/m^3^.^5^ In several states, notably Delhi, Uttar Pradesh, Bihar, and Haryana, annual mean values exceeded 125 μg/m^3^; such extreme values reduced population-wide life expectancy by an estimated 2 years. As such, while India represents 18% of the global population, it is estimated that over 26% of global disability-adjusted life years due to air pollution occur in India.^5^ Taking advantage of remote-sensing satellite technology that allows for modelling of PM2·5 across the sub-continent, other studies have demonstrated that air pollution is not solely an urban problem, with estimated total mortality due to PM2·5 being larger in non-urban areas, due to the vast non-urban population in India and high annual levels of ambient PM2·5 that occur even outside the major cities.^6^ Indeed, the seminal Global Burden of Disease study published in 2016 attributed ambient particulate matter and household air pollution as the third and fourth leading risk factors for early death and disability in India, behind only blood pressure and diabetes, and ahead of smoking, unsafe water, and iron deficiency.^7^

Children are particularly susceptible to the effects of air pollution, as they breathe more air per body weight than adults and their immature organs are unable to rapidly excrete toxins.^8^ This is especially true for paediatric respiratory diseases, as air pollution has been associated with wheezing, asthma, cough, pneumonia, and lung disease in a multitude of settings.^9^^,^^10^ Within India, the focus has largely been on indoor air pollution, given the known ill-effects of cooking with toxic fuels indoors, and numerous studies have demonstrated associations of toxic cooking and heating fuels with respiratory symptoms and diseases in childhood.^11^ Studies of ambient air pollution have largely focused on the urban setting where data collection of ambient pollution has been, until recently, more feasible. In these settings, children with high ambient air pollution exposure have been found to have worse lung function, higher rates of respiratory symptoms, and increased emergency department visits.^12^ In the Indian context, poor housing conditions, overcrowding, unsafe cooking fuel, and unsafe water have been significantly associated with increased likelihood of respiratory symptoms and infections.^13^, ^14^, ^15^

Given the importance of environmental conditions on under-5 health, we aimed to quantify the impact of these environmental conditions on respiratory illness, a key driver of childhood morbidity and mortality worldwide and in India.^16^ We hypothesised that ambient air pollution, as measured by ambient PM2·5 levels, and household air pollution, as characterised by reported household living conditions and fuel sources, would be significantly associated with severe respiratory illness (defined as cough, rapid breathing, and fever) in children younger than 5 years, while controlling for other key environmental and sociodemographic factors. Similarly, we hypothesised that other key social and environmental factors, including wealth index and housing conditions, would be associated with respiratory illnesses in this age group. Our comprehensive data sources provided health and PM2·5 data across India, allowing us to perform a population-wide analysis.

## Methods

### Study population and environmental data

We performed a retrospective, observational study, using data from the fifth iteration of India's National Family Health Survey (NFHS-5), which was carried out over two phases between June 2019 and April 2021.^17^ The NFHS-5 was a multi-round cross-sectional survey coordinated by the International Institute for Population Sciences and conducted in a representative sample of 639,699 Indian households. Using a standardised questionnaire, 724,115 eligible women aged 15–49 years were interviewed for this study. Information on several topics was collected, including basic sociodemographic characteristics; maternal health; pregnancy and postnatal care; and child health indicators. Responses from women with children born in the five years preceding the survey were reformatted using common variable names to generate a Children's Recode dataset, which was made publicly available through the Demographic Health Survey (DHS) data distribution system. The Children's Recode was used in our analysis as it contained information relevant to our target population of children younger than 5 years—including data on recent illnesses and other health parameters. Additionally, the NFHS-5 includes geographic information on each survey, in the form of ∼30,000 geographic clusters across India. Each cluster geolocation is recorded as part of the survey process and randomly displaced up to 2 km for urban clusters and 10 km for rural clusters to maintain subject privacy. We combined the NFHS-5 dataset with the Geospatial Covariate Datasets Manual to match cluster-level environmental data with each family, including altitude, rainfall, malaria prevalence, population density, urban or rural designation, region of the country, and temperature month of the interview.^18^ Additional details regarding survey design, including the two-stage stratified random sampling technique utilised, can be found in the official NFHS-5 report.^17^

### Air pollution data

PM2·5 data comes from the NASA Socioeconomic Data and Applications Center (SEDAC) Global Annual 2019 PM2·5 Grids database.^19^ This database combines aerosol optical depth retrievals from satellite algorithms and combines them with global ground-based measurements to provide annual PM2·5 estimations at a resolution of 0·01 × 0·01° (approximately 1 km × 1 km); a more detailed explanation of the methodology is provided elsewhere.^19^ Using this database, cluster-level PM2·5 exposure estimates were obtained by averaging the annual PM2·5 values within 2 km of each urban cluster geolocation and within 10 km of each rural geolocation. Distances of 2 and 10 km were chosen due to the DHS's practice of randomly displacing each urban cluster by up to 2 km and each rural geolocation by up to 10 km to maintain respondent confidentiality.

### Variables of interest

Worldwide and specifically in India, respiratory infection or pneumonia is a leading driver of preventable childhood morbidity and mortality, contributing to 13% of deaths in children aged 1 month to 5 years, more than any other single cause in this age group.^20^ As such, our dependent (outcome) dichotomous variable of interest was respiratory infection/illness—defined as answer “yes” to whether the child had suffered from cough AND fever AND rapid breathing in the previous 2 weeks. Our independent (exposure) variables of interest were chosen a priori based on data availability from the DHS and using a modified version of Bronfenbrenner's Social-Ecological framework, which considers the individual (in our analyses, we included age, sex, birthweight, current weight), micro and mesosystem (family wealth index, cooking fuels and kitchen, smoking in house, housing quality, water source, and environmental indicators including ambient PM2·5, annual rainfall, season of interview, and average temperature the month of interview), and macrosystem (urban or rural cluster location, state/union territory of cluster).^21^ Of note, we ran a sensitivity analysis where the outcome of interest was childhood anaemia, defined as measured haemoglobin as less than 11·0 g/dL per WHO standards, hypothesising that ambient air pollution would have less of an impact on childhood anaemia as compared to severe respiratory illness.^22^ See Supplement for the breakdown of how specific measures were coded.

### Statistical methods and modelling

Simple summary statistics, density plots, and choropleth maps were used to gain an initial understanding of the data. Linear associations between the independent variables of interest were examined using Pearson correlation coefficients. Bivariate analyses via bivariate logistic regression were performed between the respiratory illness (dependent variable) and independent variables of interest, as shown in Table 2. Logistic generalized additive models were employed due to the binary nature of the outcome variable (severe respiratory infection yes vs. no), which allowed us to assess the non-linear relationship between the exposure and outcome variables of interest while controlling for potential individual, social, and environmental confounders. A key advantage of generalized additive models is that they allow for the analysis of non-linear predictors; in our models, temperature and PM2·5 were incorporated as smooth non-linear terms, as both have been reported in many settings to have non-linear effects on health outcomes. Specifically, thin-plane regression splines were utilised for PM2·5 and temperature variables.^23^ We also tested multivariable models with PM2·5 as a linear predictor, which had worse overall fit than as a non-linear predictor. As such, we included only non-linear results. Additionally, generalized additive models are able to efficiently incorporate hierarchical data in large datasets, a key property given the nature of the DHS data collection.^24^ Specifically, clusters were included as random effects to account for this survey structure. Because our exposure of interest (PM2·5) is at the geographic cluster-level, we decided a priori to group by geographic cluster. Additionally, in this sample, most children (∼75%) are the only child from their household. Observations with missing data were excluded from the logistic regression models; however, due to the complete nature of DHS, this represented <2% of observations. Note that we chose not to include survey weights in our analyses, as our study question of interest does not require population average treatment effects and the sample average treatment effects obtained without weighting are more statistically efficient and allow us to analyse the study sample without modification.^25^ As a result, however, our results therefore may not accurately reflect the absolute, population-based prevalence of the key variables. Statistical analyses were performed using R (v4·2·2).^26^

### Role of the funding source

The funding agency had no role in study design, data collection, data analysis, interpretation, or writing of the manuscript.

## Results

**Fig. 1:**
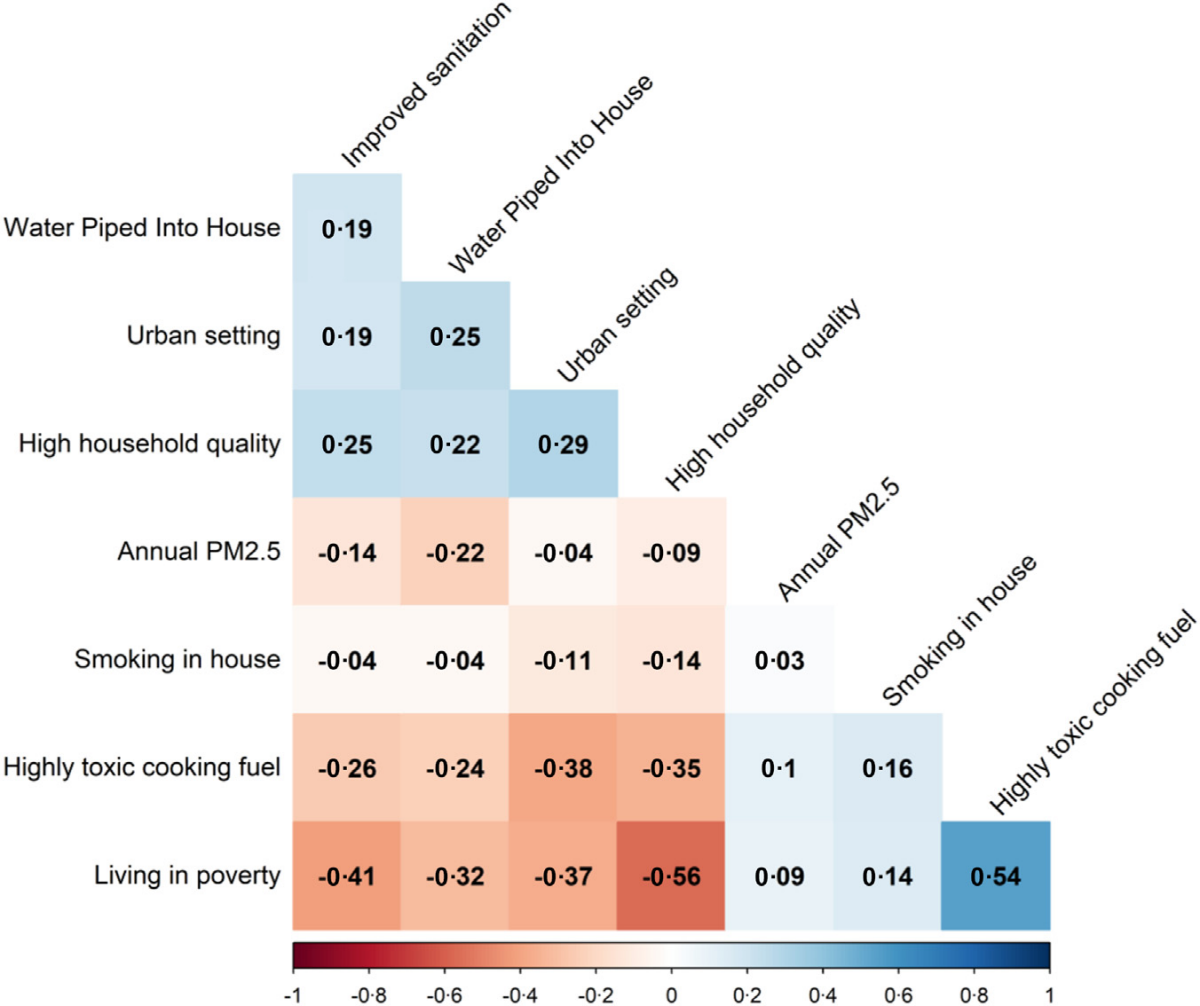
**Correlation among selected independent variables**. Correlation plot for selected social and environmental determinants of health. Red signifies highly negative correlation, white is no correlation, and blue is high positive correlation.

**Fig. 2:**
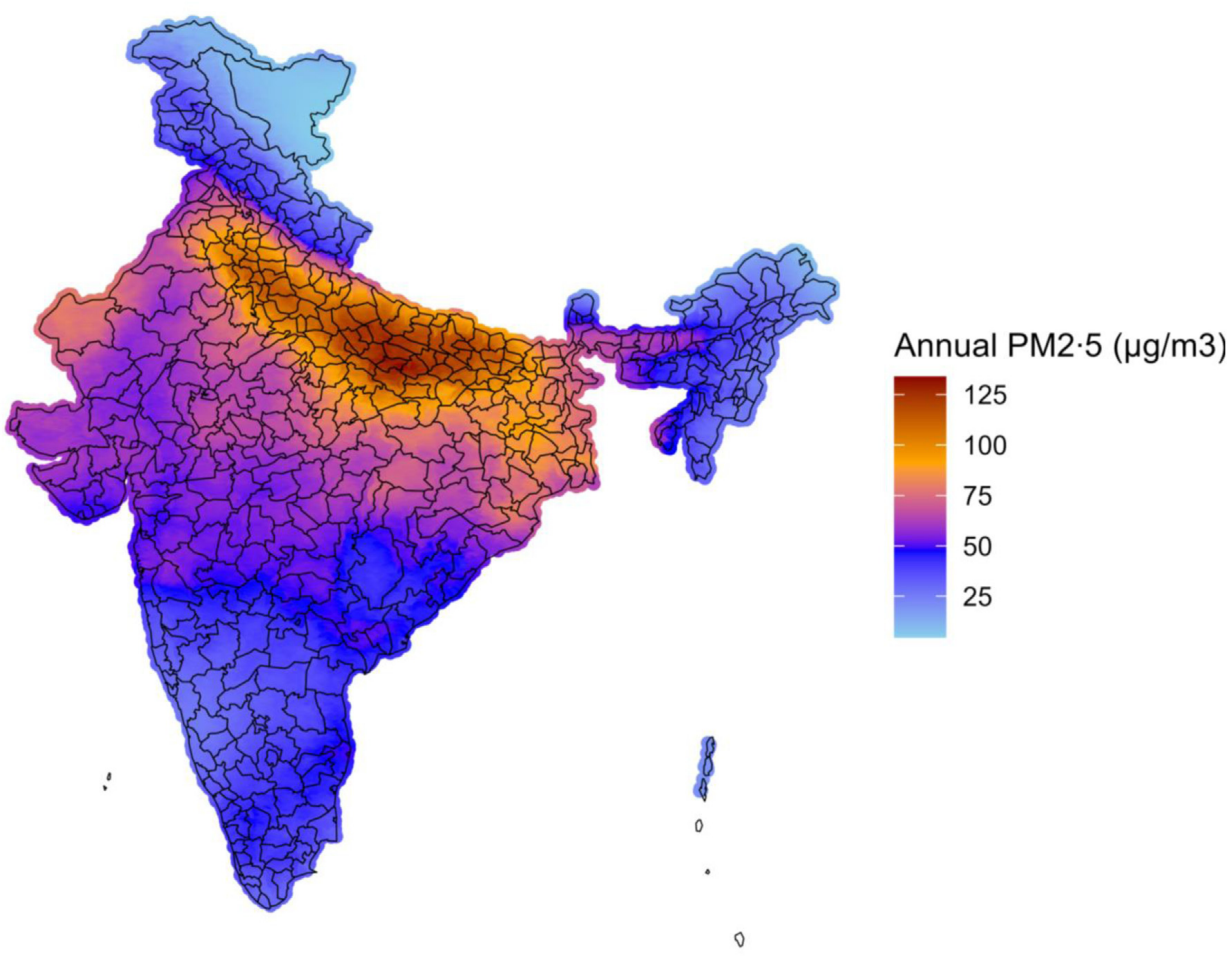
**Map of annual PM2·5 values in 1 km∗1 km grids, 2019**. Choropleth map of annual PM2·5 values across India, with data from NASA Socioeconomic Data and Applications Center (SEDAC) global annual PM2·5 data. Note the World Health Organization air quality guideline recommends annual PM2·5 < 5 µg/m^3^, and India national guidelines recommend <40 µg/m^3^.

Our sample included 224,214 children (48·3% female, median age of 30 months), representing 165,561 families from 29,757 clusters (Table 1). As previously reported, the sample comes from all regions of the country and is majority rural (79·5%). There was a high proportion of environmental factors known to be associated with poor health outcomes, including moderate to high toxicity cooking fuels (52·1%), open defecation (22%), and smoking in the house (51·1%). Fig. 1 shows the correlation coefficient among the key environmental variables (1 is perfect correlation, 0 is no correlation, and −1 is perfect inverse correlation); note that annual PM2·5 exposure is negatively correlated with measures of wealth, such as having piped water into the house (correlation coefficient −0·22), improved sanitation (−0·14), and high household quality (−0·09). Ambient PM2·5 exposure was high throughout the country (median 63·4 μg/m^3^, IQR 41·9–81·6), with the highest values in the northeast region (Fig. 2), consistent with previous studies that demonstrate air pollution levels in this region tend to be especially elevated due to a confluence of factors, including: rapid urbanisation, high population density, vehicular emissions, crop residue burning, and use of biomass for cooking.^27^ Interestingly, when looking at India as a whole, PM2·5 was slightly higher among those living in rural areas (median 63·6 (IQR 45·4–90·3 μg/m^3^) vs. urban (median 58·6 (IQR 43·7–87·5 μg/m^3^, also Fig. 1). Regarding our outcome variable, respiratory illness was reported in 7485 children (3·3%). Table 2 shows the results of our bivariate analyses. We observed significant associations between respiratory illness and various factors at the individual level (e.g., younger age of child), household level (e.g., low-quality housing material, toxic cooking fuels, open defecation), and cluster level (e.g., rural designation, annual PM2·5 levels).

**Table 1:** Individual, household, and cluster level results of total sample (N = 224,214 children under-5).

	Number or median	Percentage or interquartile range
**Child variables**		
Age of child (months)	30	(14–45)
Female (vs. male)	108,393	48·34%
Weight of child at birth (kg)	2·9	(2·5–3·0)
Weight of child currently (kg)	10·8	(8·4–13·1)
**Outcome variable**		
Respiratory Infection/Illness	7485	3·34%
**Cluster level variables**		
Annual PM2·5 Levels (μg/m^3^)	62·655	(45·01–89·87)
Region of country		
North	41,775	18·63%
Central	57,460	25·63%
East	43,271	19·3%
Northeast	33,199	14·81%
West	19,951	8·9%
South	28,562	12·74%
Urban designation (vs. rural)	45,884	20·46%
**Household variables**		
Cooking fuel toxicity		
Moderate to high toxicity	116,928	52·15%
Non-toxic or Low-toxicity	96,427	43·01%
Other	10,863	4·84%
Health insurance coverage for ≥1 household member (vs. no/don't know)	87,834	39·17%
High quality housing material (vs. low quality)	99,331	44·3%
Household has a separate room as a kitchen, yes (vs. no)	115,776	66·65%
Mother has not completed primary schooling (vs. has completed primary schooling)	77,256	34·46%
Number of people living in household	6	(4–7)
Sanitation or toilet facilities		
Improved sanitation	166,068	75·06%
Open defecation	48,985	22·14%
Unimproved sanitation	6183	2·79%
Smoking of tobacco in house: yes (vs. no)	114,461	51·05%
Water source: piped into house (vs. not piped into house)	66,346	29·59%
Wealth index poor (vs. not poor)	112,406	50·13%

Children under-5: children younger than 5 years.

Respiratory Infection/Illness: Demographic Health Survey (DHS), has child suffered from cough AND fever AND short rapid breaths/difficulty breathing in last two weeks (yes vs. no)?

Cooking fuel toxicity: electricity, liquid petroleum gas, natural gas, and biogas are classified as non-toxic or low-toxicity, all else is moderate to highly toxic.

High quality housing material includes fabricated material, such as ceramic tiles, brick, cement, polished stone, marble, etc. All else is low quality.

Sanitation/toilet facilities: As classified by DHS, improved is flush toilet or modified pit latrine, unimproved is open pit latrine or bucket, open is non toilet facility/location.

Urban designation: As classified by DHS, including large cities, small cities, and towns (other urban areas); all else rural.

Wealth Index: Composite measure of household's cumulative living standard, divided in quintiles by DHS, and dichotomized into poor (bottom two quintiles) vs. not poor.

**Table 2:** Results of bivariate analyses, outcome of respiratory infection.

Independent variables	Respiratory infection/illness			
	Estimate	Standard error	Odds ratio	p-value
**Cluster level variables**				
Altitude (m)	<0·001	<0·001	1·000	<0·001
Annual PM2·5 Levels (2019, μg/m^3^)	0·008	<0·001	1·008	<0·001
Annual Rainfall (2020, mm per year)	0·001	<0·001	1·001	<0·001
Malaria Prevalence (2020, Plasmodium falciparum parasite rate)	−13·389	1·768	<0·001	<0·001
Population Density (persons per km^2^)	<0·001	<0·001	1·000	<0·001
Region of country (Ref: North)				
Central	0·087	0·039	1·091	0·027
East	0·736	0·037	2·088	<0·001
Northeast	0·025	0·045	1·025	0·58
West	0·083	0·052	1·087	0·109
South	0·099	0·046	1·104	0·033
Urban designation (vs. rural)	−0·209	0·031	0·811	<0·001
**Household variables**				
Average temperature (Celsius, month of interview)	0·01	0·002	1·010	<0·001
Cooking fuel toxicity (Ref: Moderate to high toxicity)				
Non-toxic or Low-toxicity	−0·177	0·025	0·838	<0·001
Other	0·066	0·053	1·068	0·21
Health insurance coverage for ≥1 household member	−0·04	0·024	0·961	0·101
High quality housing material (vs. low quality)	−0·224	0·024	0·799	<0·001
Household has a separate room as a kitchen, yes	−0·245	0·028	0·783	<0·001
Mother has not completed primary schooling	0·097	0·024	1·102	<0·001
Number of people living in household	0·002	0·004	1·002	0·654
Sanitation or toilet facilities (Ref: Improved sanitation)				
Open defecation	0·231	0·027	1·260	<0·001
Unimproved sanitation	0·109	0·071	1·115	0·124
Season of Interview (Ref: Monsoon)				
Post-Monsoon	−0·105	0·032	0·900	0·001
Summer	−0·39	0·041	0·677	<0·001
Winter	−0·209	0·03	0·811	<0·001
Smoking of tobacco in house: yes (vs. no)	0·05	0·024	1·051	0·032
Water source: piped into house	−0·302	0·028	0·739	<0·001
Wealth index poor (vs. not poor)	0·267	0·024	1·306	<0·001
**Child variables**				
Age of child (months)	−0·009	0·001	0·991	<0·001
Female (vs. male)	−0·14	0·024	0·869	<0·001
Weight of child at birth (kg)	<0·001	<0·001	1·000	0·002
Weight of child currently (kg)	0·055	0·016	1·057	0·001

Respiratory Infection/Illness: Demographic Health Survey (DHS), has child suffered from cough AND fever AND short rapid breaths/difficulty breathing in last two weeks (yes vs. no)?

Cooking fuel toxicity: electricity, liquid petroleum gas, natural gas, and biogas are classified as non-toxic or low-toxicity, all else is moderate to highly toxic.

High quality housing material includes fabricated material, such as ceramic tiles, brick, cement, polished stone, marble, etc. All else is low quality.

Population Density: UN adjusted population density (number of people per square km).

Sanitation/toilet facilities: As classified by DHS, improved is flush toilet or modified pit latrine, unimproved is open pit latrine or bucket, open is non toilet facility/location.

Urban designation: As classified by DHS, including large cities, small cities, and towns (other urban areas); all else rural.

Wealth Index: Composite measure of household's cumulative living standard, divided into quintiles by DHS, and dichotomized into poor (bottom two quintiles) vs. not poor.

Estimates and p-values obtained via bivariate logistic regression, where dependent variable was severe respiratory infection.

p-values <0·05 were considered as statistically significant.

**Fig. 3:**
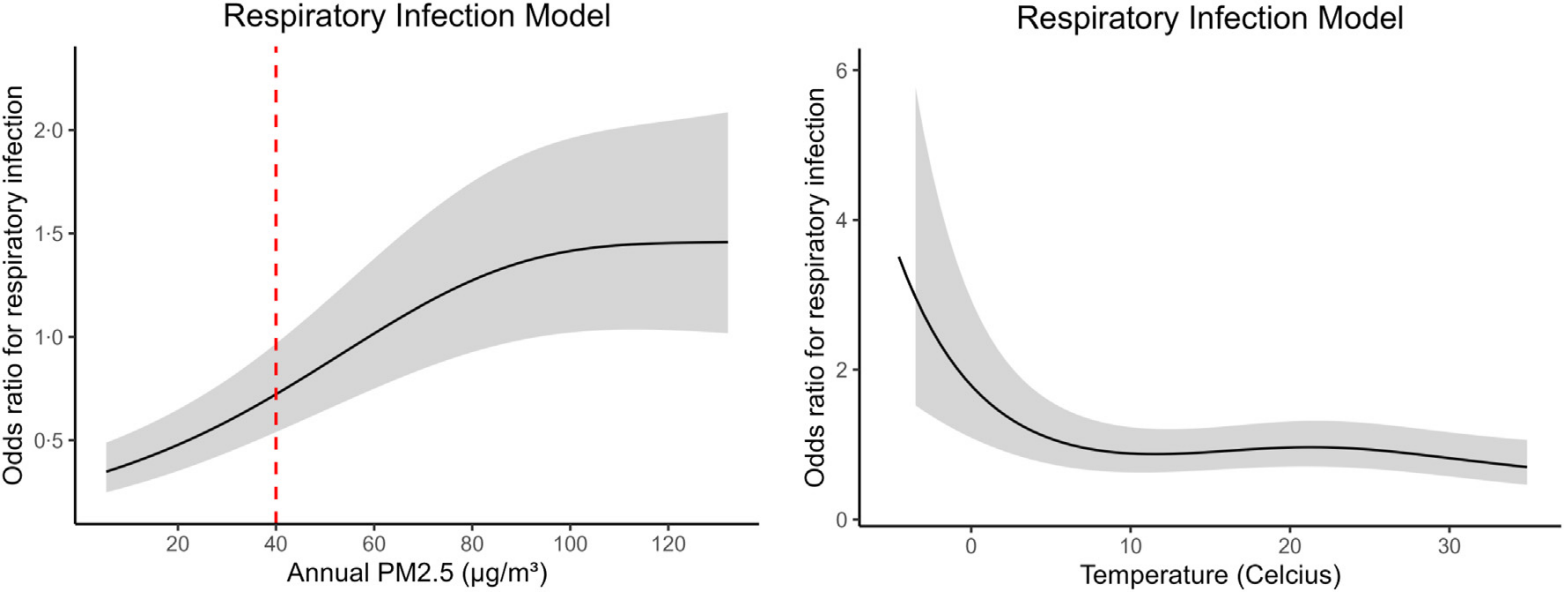
**Smoothed terms of generalized additive model**. Smooth terms of annual PM2·5 levels (left) and mean temperature month of interview (right) from the generalized additive models, with outcome of reported respiratory infection. The graphs represent the nonlinear effect of annual PM2·5 and temperature on the probability of respiratory infections. Note that a steeper slope (as seen in the respiratory infection model from PM2·5 ∼20 to 80 µg/m^3^) indicates a stronger effect, and the grey bands indicate 95% confidence intervals. Dashed vertical line indicates the Indian PM2·5 annual standard of 40 µg/m^3^.

Table 3 shows the results of the multivariable generalized additive models. We observe significant associations of cluster-level (e.g., annual rainfall), household-level (e.g., season household was interviewed), and child-level (e.g., age, sex) independent variables with likelihood of respiratory illness. Fig. 3 presents the results of the smoothed (non-linear) terms of annual PM2·5 levels and temperature**.** The plots reflect the nonlinear relationship between the variables and the risk of severe respiratory illness, while controlling for socioeconomic, environmental, and individual health factors. The x-axis shows annual PM2·5 levels and temperature the month of interview, and the y-axis shows the estimated log-odds of the severe respiratory illness at that value, as compared to the median annual PM2·5 level (∼60 μg/m^3^) and median temperature the month of interview. The direction and steepness of the curve inform us about the relationship between the independent and dependent variables. Specifically, a steeper positive slope signifies that an increase in PM2·5 levels leads to a more substantial rise in infection risk, while a flatter slope suggests that the change in risk is smaller or negligible, at that PM2·5 level. The gray bands demarcate the 95% confidence intervals for the smoothed terms. In Fig. 3, an observable and statistically significant effect (p < 0·001) is evident: as annual PM2·5 levels increase, the likelihood of severe respiratory illness rises as well. This relationship is most pronounced within a range of approximately 0–60 μg/m^3^ for PM2·5. In other words, the consistent positive slope of the PM2·5 graph indicates that as the level of PM2·5 increases, the predicted log-odds of having a severe respiratory illness also continues to increase, and the graph being steepest from 0 to 60 μg/m^3^ demonstrates that the rate of increase in risk is highest for this interval. We see similar results in our sub-analyses by region, showing the predicted odds of respiratory infection increases as PM2·5 increases in five of six regions, indicating air pollution is a significant problem throughout the country. It is worth noting that the western region (western part of India) is the outlier, demonstrating a flat shape; however, the western region has the fewest observations, widest confidence intervals, and thus likely underpowered to detect a significant association (Supplemental Figure).

**Table 3:** Results of generalized additive model.

Independent variables	Respiratory infection/illness			
	Estimate	Standard error	Odds ratio	p-value
**Cluster level variables**				
Altitude (m)	0·010	0·03	1·010	0·746
Annual PM2·5 Levels (2019, μg/m^3^)	Smooth term–see graph			
Annual Rainfall (2020, mm per year)	**0·115**	**0·023**	**1·122**	**<0·001**
Malaria Prevalence (2020, *Plasmodium falciparum* parasite rate)	**−0·133**	**0·026**	**0·875**	**<0·001**
Urban designation (vs. rural)	−0·059	0·051	0·943	0·247
**Household variables**				
Average temperature (month of interview)	Smooth term–see graph			
Cooking fuel toxicity (Ref: Moderate to high toxicity)				
Non-toxic or Low-toxicity	−0·017	0·042	0·983	0·69
Other	0·001	0·079	1·001	0·989
High quality housing material (vs. low quality)	−0·073	0·04	0·930	0·071
Sanitation or toilet facilities (Ref: Improved sanitation)				
Open defecation	0·047	0·043	1·048	0·271
Unimproved sanitation	−0·079	0·1	0·924	0·429
Season of Interview (Ref: Monsoon)				
Post-Monsoon	**−0·162**	**0·057**	**0·850**	**0·005**
Summer	**−0·515**	**0·072**	**0·598**	**<0·001**
Winter	**−0·346**	**0·066**	**0·708**	**<0·001**
Smoking of tobacco in house: yes (vs. no)	0·022	0·034	1·022	0·521
Water source: piped into house	−0·063	0·043	0·939	0·138
Wealth index poor (vs. not poor)	0·037	0·048	1·038	0·443
**Child variables**				
Age of child (months)	**−0·189**	**0·025**	**0**·**828**	**<0·001**
Female (vs. male)	**−0·141**	**0·032**	**0**·**868**	**<0·001**
Weight of child currently (kg)	−0·040	0·027	0·961	0·135
Weight of child at birth (kg)	−0·010	0·016	0·990	0·525
Total number of observations	145,675			

Respiratory Infection/Illness: Demographic Health Survey (DHS), has child suffered from cough AND fever AND short rapid breaths/difficulty breathing in last two weeks (yes vs. no)?

Cooking fuel toxicity: electricity, liquid petroleum gas, natural gas, and biogas are classified as non-toxic or low-toxicity, all else is moderate to highly toxic.

High quality housing material includes fabricated material, such as ceramic tiles, brick, cement, polished stone, marble. All else is low quality.

Sanitation/toilet facilities: As classified by DHS, improved is flush toilet or modified pit latrine, unimproved is open pit latrine or bucket, open is non toilet facility or location.

Urban designation: As classified by DHS, including large cities, small cities, and towns (other urban areas); all else rural.

Wealth Index: Composite measure of household's cumulative living standard, divided in quintiles by DHS, and dichotomized into poor (bottom two quintiles) vs. not poor.

Estimates and p-values obtained via generalized additive multivariable logistic regression, dependent variable was respiratory infection. p-values <0·05 were considered as statistically significant (in bold).

Models include random effects for cluster.

To test the validity of our models, we ran sensitivity analyses, where the outcome of interest was childhood anaemia rather than respiratory illness. As described above, we observe an increasing effect of annual PM2·5, whereas this effect is not observed for the anaemia model (Supplemental Figure). The stronger correlation observed between annual PM2·5 levels and respiratory illness, as compared with under-5 anaemia, aligns with our physiological understanding, as airborne particulate matter is known to aggravate the respiratory system, increasing susceptibility to infections. Conversely, the lack of a similar relationship in the anaemia model is anticipated, given that anaemia is less directly influenced by air quality. As such, these results add validity to our models, underscoring the specificity of the modelled health impacts linked to air pollution and adding evidence of ambient air pollution being a driver of respiratory illness in young children rather than simply an association.

## Discussion

In this population-wide analysis of 224,214 children under 5 throughout India, our results confirm the previous findings of high ambient air pollution levels throughout India, with families living in rural clusters and in poverty having higher average exposure to ambient PM2·5. Furthermore, we observed that ambient PM2·5 was significantly associated with respiratory illness in children under 5 years of age, both before and after controlling for key environmental and socioeconomic factors associated with childhood health, consistent with the hypothesis that ambient PM2·5 is a significant driver of respiratory illness and infection in this population.

Using generalized additive models, we observe a non-linear relationship between PM2·5 and respiratory illness, showing an increasing likelihood of respiratory illness that plateaus at approximately 100 μg/m^3^ (Fig. 3). Within the Indian context, the shape of this graph has several important implications. First, our study adds to a body of literature suggesting ambient air pollution has harmful effects in many health settings; here, we show that children under-5 may be at particularly high risk. Our findings complement prior studies on ambient PM2·5 in India; previous studies using remote-sensing data have demonstrated that early life PM2·5 exposure is associated with poor growth and overall under-5 mortality in India.^28^, ^29^, ^30^ With the median household exposure to PM2·5 at 63 μg/m^3^ in our sample, considerably above both WHO (5 μg/m^3^) and Indian (40 μg/m^3^) target levels, improving ambient air quality would likely confer significant health benefits to young children. Indeed, in 2019 the Indian Government launched the National Clean Air Programme, with the goal of reducing PM10 and PM2·5 by 20–30% by 2024.^31^ This initiative marks an important step towards reducing morbidity attributed to air pollution; however, our results show a steep upward slope at India's PM2·5 standard of 40 μg/m^3^, suggesting that this level still does not represent a safe concentration and even stricter air pollution standards would have significant health benefits.

We observe a plateau at 100 μg/m^3^; this similar plateau at extremely high pollutant levels has been observed in several other studies in different settings, though one study of PM2·5 in India showed steeper associations at higher PM2·5 concentrations.^32^ This plateau is also observed in the sub-group analysis, in which the Central region has both extremely high values of PM2·5 throughout and a relatively flat (albeit still positively sloped) graph. There are mechanisms that could plausibly explain this plateau, such as pollution avoidance behaviours at high levels, the impact of other, unmeasured pollutants, or simply spurious associations due to relatively smaller numbers of patients exposed to the most extreme PM2·5 values. Additionally, our outcome of interest is limited to reported respiratory symptoms; it is possible that we would observe increasing likelihood and/or severity of other health outcomes at these higher values. Furthermore, the pathophysiology of the interaction between PM2·5 and the respiratory system provides reason to suspect that increasing PM2·5 exposure levels would continue to cause increasing, as opposed to plateauing, harm. Considering this broader context, we cannot conclude that 100 μg/m^3^ represents a threshold above which there are no or few additional harms.

There is a strong link between household air pollution and respiratory infection and illness among children. In India, household air pollution arises from the burning solid fuels such as coal, wood, and biomass for cooking and heating purposes, which releases harmful pollutants into the air. One study conducted in rural areas of West Bengal found that children exposed to high levels of household air pollution were at a higher risk of developing respiratory infection than those with lower levels of exposure.^33^ Another study conducted in the urban slums of Delhi found that children living in households with higher levels of household air pollution had a higher incidence of respiratory infection than those living in households with lower levels of pollution.^13^ A systematic review and meta-analysis of studies conducted in India also found a significant association between household air pollution and respiratory infection among children. The review included 15 studies that investigated the association between household air pollution and respiratory infection, and the results showed that children living in households with higher levels of household air pollution had a 40% higher risk of developing a respiratory infection than those living in households with lower levels of household air pollution.

Our study did not uncover a significant association between cooking fuel type and respiratory illness. However, it has been estimated that domestic fuel burning contributes up to 30–40% of ambient PM2·5 in India.^34^ Considering that household air pollution significantly contributes to ambient air pollution in low and middle income countries such as India, our models may under-estimate the true impact of toxic fuel sources within individual households. In other words, although classified as “ambient,” the PM2·5 findings in this study are also partially attributable to household air pollution. In response to the childhood health implications of household air pollution, several interventions have been implemented in India. These interventions include the promotion of clean cooking technologies and behavioural changes to reduce household air pollution exposure. While these interventions have shown promise in reducing household air pollution exposure and improving children's health outcomes, more research is needed to fully understand their effectiveness and scalability. Overall, the evidence suggests that household air pollution is a significant risk factor for respiratory infection among children in India, and addressing this issue is critical for improving children's health and reducing the burden of disease in the country.

Our study has limitations worth mention. This is a retrospective study and causality cannot be directly inferred from its findings alone. However, our analysis reinforces a growing body of literature demonstrating the importance of clean air in human health, growth, and development.^8^ From an air pollution standpoint, we have chosen PM2·5 as our pollutant of interest given data availability; although PM2·5 is arguably the most thoroughly researched pollutant with regards to human health, it is important to recognise there are other pollutants, such as nitrogen dioxide, ozone, and carbon monoxide, that are hazardous to health and are in many cases correlated with PM2·5 levels.^35^ Thus, it may be a mixture of air pollutants, as opposed to solely PM2·5, causing the effects observed. Additionally, our ambient air pollution measure is obtained at the cluster level, as opposed to the individual household level. Although clusters are relatively small geographic areas, which limits the measurement error, this source of error still exists in this study. Furthermore, SEDAC data is limited to 2019, which represents another source of measurement error, as the household surveys took place from 2019 to 2021. Additionally, the PM2·5 variable is annual rather than short-term exposure, another source of measurement error. If this measurement error is randomly distributed, this source of error will bias our results towards the null, meaning our results may underestimate the true effect. The outcomes of interest, respiratory illness, is based on results of a large-scale survey and thus subject to recall and social desirability biases. There is reason to suspect that children with asthma or atopy (or children with a family history of asthma or atopy) could be even more susceptible to the effects of air pollution, as has been shown in other settings. However, this data is not reliably available (especially family history, given that women predominantly serve as caregivers and are the primary respondents in this survey). Furthermore, we did not have data on actual indoor pollutants; instead, we used proxy measures (such as cooking fuels, housing design and quality), which, while utilised in other settings and studies in India, are not actual measures of indoor pollution.^36^ To account for this, we have controlled for various individual, household, and community measures that may be correlated with these proxy measures and our outcome of interest in our multivariable model. Due to data limitations, there are other key, unmeasured confounders that may bias our results. For example, access to healthcare and other measures of socioeconomic status (such as standing in community, social networks) are known to be associated with health outcomes. Additionally, other environmental factors, such as unsafe drinking and bathing water, heat and cold exposure, neighbourhood safety, and noise or light pollution, are associated with negative health outcomes.^37^ While we included proxy measures for several of these variables (e.g., housing quality, usual water source), more detailed, precise measurement and analysis of these key social and environmental determinants of health is needed in future studies.

Collectively, the significant associations observed between ambient and household air pollution sources support the hypothesis that these environmental factors are key drivers of respiratory illness in children younger than 5 years. As pneumonia is the leading cause of death in children 1 month to 5 years of age and India is estimated to have the highest number of under-5 deaths globally,^16^^,^^20^ these findings suggest that initiatives and policies aimed at minimising exposure to both ambient and household air pollution could have significant health benefits for this high-risk group. Furthermore, concerted efforts at improving housing and sanitation conditions among the highest-risk families might have important health benefits for children under 5 years of age and advance India towards the WHO Sustainable Development Goal^38^ of ending preventable deaths of children younger than 5 years.

## Contributors

PEG: conceptualisation, data analysis, and writing–original draft. NT: conceptualisation, writing, and writing - editing. SY: data analysis (supervision), writing, and editing. DS: supervision and writing–editing. JS was responsible for conceptualisation, supervision, and writing–editing. PEG and NT: directly accessed and verified the underlying data reported in the manuscript. All authors edited and approved the final version of the manuscript.

## Data sharing statement

The data for this study come from publicly available data, which can be accessed upon request from the relevant organisations. The code used for data analysis will be uploaded to https://github.com/pegeorge for use and reference upon publication.

## Editor note

The Lancet Group takes a neutral position with respect to territorial claims in published maps and institutional affiliations.

## Declaration of interests

PEG was supported by the National Institutes of Health (NIH T-32-HL139443-3) training grant. All authors declare no other conflicts of interest.

## Appendix A Supplementary data

Supplementary material clean
