# Supplementary material for: Impact of ambient air pollution and socio-environmental factors on the health of children younger than 5 years in India: a population-based analysis
Source: Lancet Reg Health Southeast Asia. 2023 Dec 2;20:100328. doi: 10.1016/j.lansea.2023.100328 (PMC10731218; doi:10.1016/j.lansea.2023.100328)
Supplement: Supplementary material clean [file mmc1.docx]

**Supplementary material - Variable overview**

Outcome variables

Respiratory Infection: DHS Survey, has child suffered from cough AND fever AND short rapid breaths/difficulty breathing in last two weeks (yes versus no)?

Anaemia: DHS biomarker data, defined as haemoglobin ≤ 11 grams/dL, in accordance with WHO standards.

Independent variables

*Cluster level*

*Of note, all cluster level variables, except for PM2·5, are obtained from the DHS Geospatial Covariate Dataset, India, 2019-2021.*

Annual PM2·5: PM2·5 data comes from the NASA Socioeconomic Data and Applications Center (SEDAC) Global Annual PM2·5 Grids database. This database combines aerosol optical depth retrievals from satellite algorithms and combines them with global ground-based measurements to provide annual PM2·5 estimations in µg/m^3^ at a resolution of 0.01 x 0.01 degrees (approximately 1 km x 1 km); a more detailed explanation of the methodology is provided elsewhere.^29^ Using this database, cluster-level PM2·5 exposure estimates were obtained by averaging the annual PM2·5 values within 2 km of each urban cluster geolocation and within 10 km of each rural geolocation. 2 and 10 km were chosen because each urban cluster geolocation is randomly displaced by up to 2 km and each rural geolocation is displaced by up to 10 km by the DHS as a means of maintaining respondent confidentiality.

Altitude of cluster midpoint in meters.

Malaria Prevalence: malaria prevalence in cluster in 2020, by *Plasmodium falciparum* parasite rate.

Population Density: average UN adjusted population density (number of people per square km). See reference for more details.^1^

Rainfall: annual rainfall in cluster in 2020.

Urban designation: As classified by DHS, urban areas are classified into large cities (capital cities and cities with over 1 million population), small cities (population over 50,000), and towns (other urban areas), and all rural areas are assumed to be countryside.

*Household*

Cooking fuel toxicity: electricity, liquid petroleum gas, natural gas, and biogas are classified as non-toxic/low-toxicity, all else, including kerosene, coal, charcoal, wood, straw/shrubs/grass, agricultural crops, animal dung, are classified as moderate to highly toxic.

Health insurance: Answer of yes to whether at least one member of the household was covered by health insurance at time of interview.

High quality housing material includes fabricated material, such as ceramic tiles, brick, cement, polished stone, polished wood, marble, carpet, granite. All else, including mud, clay, earth, sand, dung, raw wood, palm, bamboo, is low quality.

Number of people living in household at time of survey.

Sanitation or toilet facilities: As classified by DHS, improved is flush toilet or modified pit latrine, or composting toilet; unimproved is open pit latrine, bucket, or hanging toilet; open is no toilet facility or set location, including bush, field, river, sea, or lake.

Season of Interview: Divided into Monsoon (June – September), post-Monsoon (October – November), Summer (March – May), and Winter (December – February).

Smoking of tobacco in house: Divided into yes (answer of daily, weekly, monthly, or less than once per month) versus no (never).

Temperature (Celsius): Average temperature of the cluster the month of the household interview.

Water source: Considered piped water if piped into the family’s dwelling or yard/plot, all else non-piped water.

Wealth Index: Composite measure of household's cumulative living standard, which considers assets, housing materials, and types of water access and sanitation facilities. The index is divided into quintiles by DHS, and then subsequently we dichotomized into poor (bottom two quintiles) vs not poor.

*Child*

Age of child (months)

Sex of child

Weight of child at birth (kilograms)

Weight of child currently (kilograms)
