# Supplementary material for: Impact of ambient air pollution and socio-environmental factors on the health of children younger than 5 years in India: a population-based analysis
Source: Lancet Reg Health Southeast Asia. 2023 Dec 2;20:100328. doi: 10.1016/j.lansea.2023.100328 (PMC10731218; doi:10.1016/j.lansea.2023.100328)
Supplement: Supplementary Figures [file mmc2.pptx]

## Slide 1
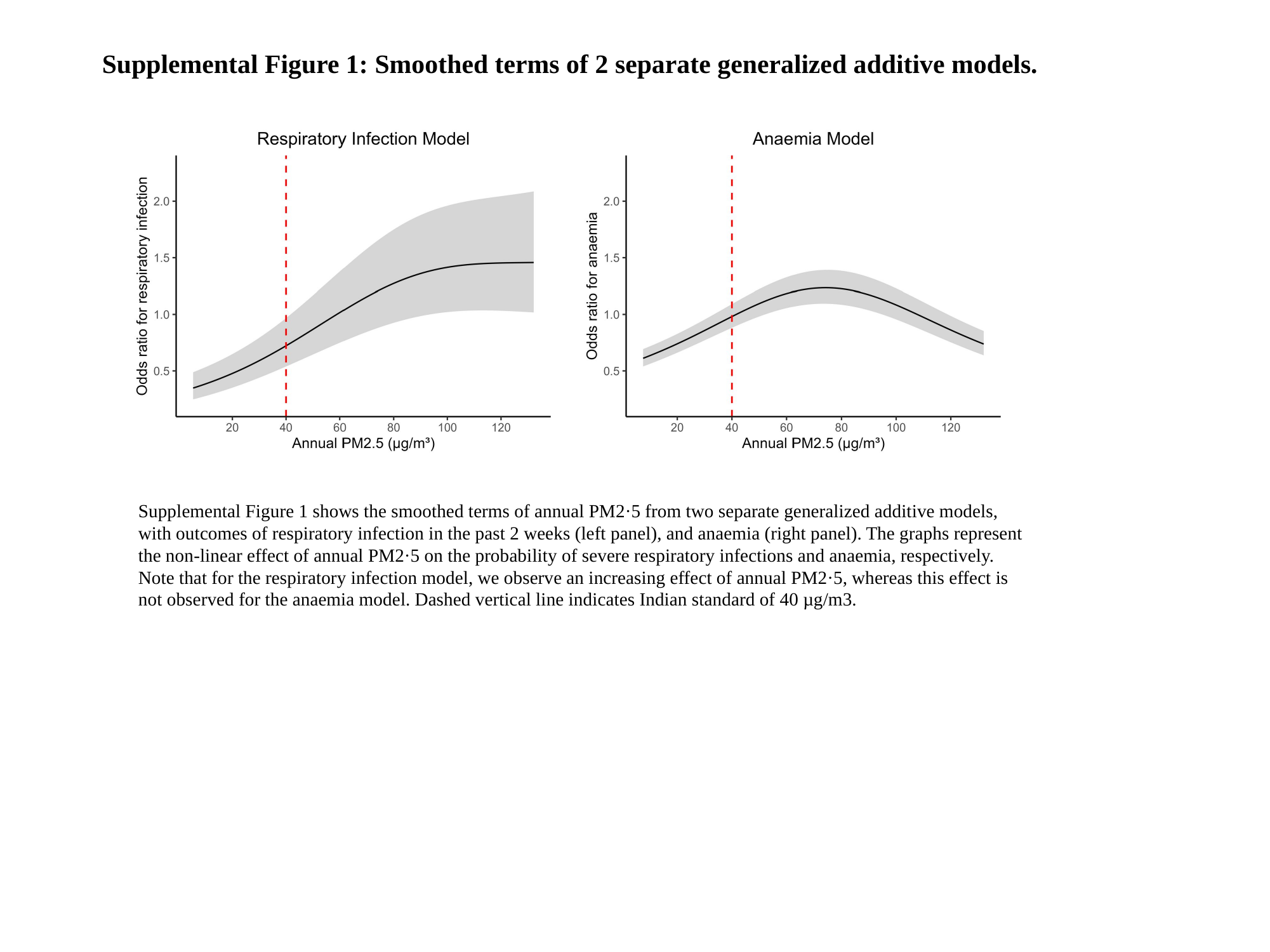

Supplemental Figure 1: Smoothed terms of 2 separate generalized additive models.
Supplemental Figure 1 shows the smoothed terms of annual PM2·5 from two separate generalized additive models, with outcomes of respiratory infection in the past 2 weeks (left panel), and anaemia (right panel). The graphs represent the non-linear effect of annual PM2·5 on the probability of severe respiratory infections and anaemia, respectively. Note that for the respiratory infection model, we observe an increasing effect of annual PM2·5, whereas this effect is not observed for the anaemia model. Dashed vertical line indicates Indian standard of 40 µg/m3.

## Slide 2
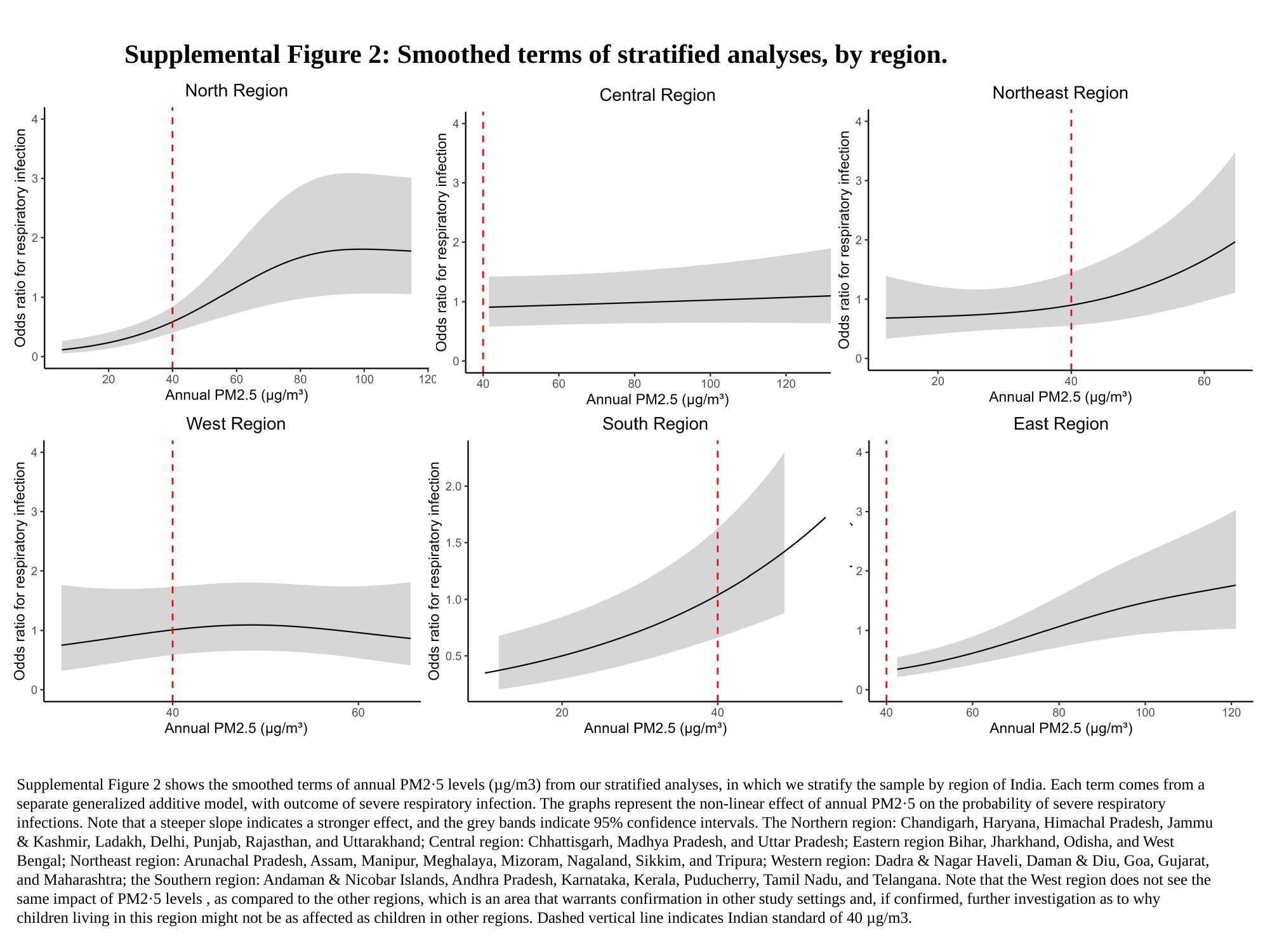

Supplemental Figure 2: Smoothed terms of stratified analyses, by region.
Supplemental Figure 2 shows the smoothed terms of annual PM2·5 levels (µg/m3) from our stratified analyses, in which we stratify the sample by region of India. Each term comes from a separate generalized additive model, with outcome of severe respiratory infection. The graphs represent the non-linear effect of annual PM2·5 on the probability of severe respiratory infections. Note that a steeper slope indicates a stronger effect, and the grey bands indicate 95% confidence intervals. The Northern region: Chandigarh, Haryana, Himachal Pradesh, Jammu & Kashmir, Ladakh, Delhi, Punjab, Rajasthan, and Uttarakhand; Central region: Chhattisgarh, Madhya Pradesh, and Uttar Pradesh; Eastern region Bihar, Jharkhand, Odisha, and West Bengal; Northeast region: Arunachal Pradesh, Assam, Manipur, Meghalaya, Mizoram, Nagaland, Sikkim, and Tripura; Western region: Dadra & Nagar Haveli, Daman & Diu, Goa, Gujarat, and Maharashtra; the Southern region: Andaman & Nicobar Islands, Andhra Pradesh, Karnataka, Kerala, Puducherry, Tamil Nadu, and Telangana. Note that the West region does not see the same impact of PM2·5 levels , as compared to the other regions, which is an area that warrants confirmation in other study settings and, if confirmed, further investigation as to why children living in this region might not be as affected as children in other regions. Dashed vertical line indicates Indian standard of 40 µg/m3.

## Slide 3
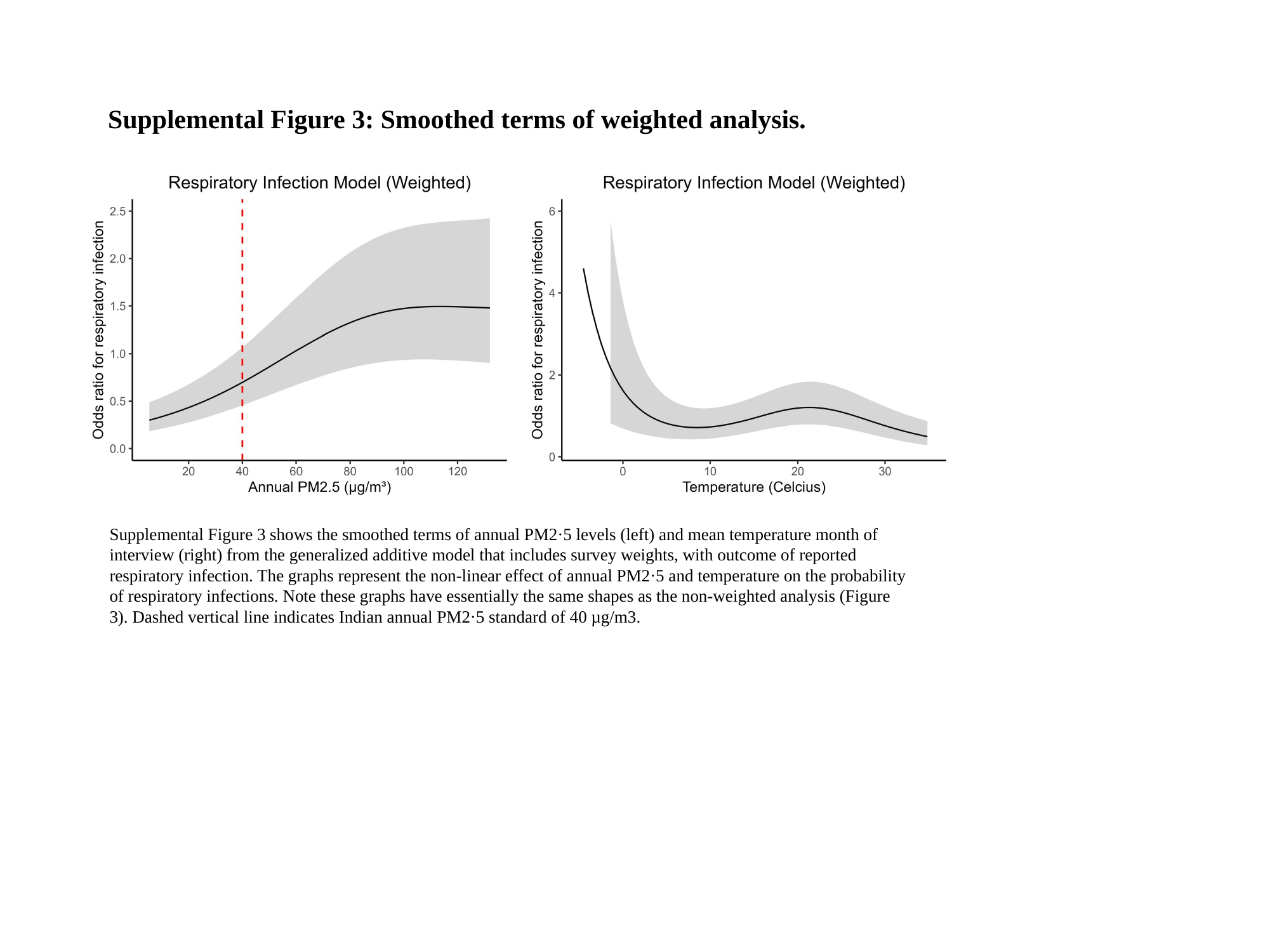

Supplemental Figure 3: Smoothed terms of weighted analysis.
Supplemental Figure 3 shows the smoothed terms of annual PM2·5 levels (left) and mean temperature month of interview (right) from the generalized additive model that includes survey weights, with outcome of reported respiratory infection. The graphs represent the non-linear effect of annual PM2·5 and temperature on the probability of respiratory infections. Note these graphs have essentially the same shapes as the non-weighted analysis (Figure 3). Dashed vertical line indicates Indian annual PM2·5 standard of 40 µg/m3.
